# Zeolite-Supported Aggregate as Potential Antimicrobial Agents in Gypsum Composites

**DOI:** 10.3390/ma15093305

**Published:** 2022-05-05

**Authors:** Magdalena Król, Justyna Syguła-Cholewińska, Tomasz Sawoszczuk

**Affiliations:** 1 Faculty of Materials Science and Ceramic, AGH University of Science and Technology, 30 Mickiewicza Av., 30-059 Krakow, Poland; 2 Microbiology Department, Institute of Quality Sciences and Product Management, College of Management and Quality Sciences, Cracow University of Economics, 27 Rakowicka, 31-510 Krakow, Poland; sygulaj@uek.krakow.pl (J.S.-C.); tomasz.sawoszczuk@uek.krakow.pl (T.S.)

**Keywords:** zeolite, porous filler, gypsum, ion-exchange, antibacterial test

## Abstract

Relatively easy treatment of glass aggregates can lead to the formation of a highly porous zeolite aggregate. This study focuses on the possibility of using such an aggregate as an active additive to a gypsum binder. The physical properties of hardened gypsum composites with zeolite fillers doped with various metal ions (Ni^2+^, Cu^2+^, and Zn^2+^) have been compared. In addition to studies of the basic physical properties of the composites, structural and microstructural studies as well as antimicrobial tests were performed. It was found that the parameters of the composites with the addition of various ions do not differ significantly from the reference but modifies the microstructure. Among other things, the ions analyzed reduce the microporosity of gypsum composites. Using all aggregates, a product with adequate strength (above 2 MPa) and thermal conductivity (about 0.35 W/m·K) appropriate for typical lightweight gypsum composites can be obtained. The bacteriostatic effect of formulations with copper and zinc against *Escherichia coli* and with copper against *Staphylococcus aureus* was found.

## 1. Introduction

The construction sector is an example where harmful microorganisms not only can lead to material loss but also can have a negative impact on human health. Colonies of mold and bacteria that form on the surface of coatings and paints contribute to the formation of ‘sick building syndrome’, which has gathered growing concern among experts. Trends in modern construction (e.g., tight carpentry, high humidity, and wood-based building composite), which result from energy savings requirements, are undeniably conducive to mold formation. Biodeterioration leads to the destruction of coatings and mortar, as well as basic building materials. However, in recent years, the tendency to reduce the content of volatile organic compounds (VOC [1], such as phenol compounds or chloroacetamide) in many products has been observed, leading to the formation of a large group of paints based on the aqueous solvent. The aquatic environment is conducive to the growth of microorganisms. To inhibit or prevent microbial growth in materials and painting, suitable biocides are used. Their mechanism of action is based on the disruption of biological processes in the cells of microorganisms (e.g., by reducing the supply of nutrients, leading to cell death) and the inhibition of their ability to reproduce. Individual chemical companies have developed and patented a wide variety of biocides based primarily on a broad spectrum of organic compounds that effectively influence the life processes of microorganisms. These include, among others, phenols or aliphatic hydrocarbons or heterocyclic compounds containing sulfur or nitrogen atoms in their composition. Particularly noteworthy is inorganic zeolite, which slowly releases silver ions. The bactericidal activity of both elemental silver and Ag^+^ ions has long been known [2,3,4,5]. However, a new approach in this work consists of looking at other ions as potential additives to zeolite that could be used as a composite filler. Biocidal formulations containing zeolites do not cause allergic reactions in humans; they are nontoxic, odorless, and considered to be safe for the environment.

Owing to isomorphous substitution, the aluminosilicate framework carries a permanent negative charge balanced by exchangeable cations. Furthermore, zeolites tend to adsorb water molecules, which hydrate exchangeable cations. Because of their porous structure, zeolites are distinguished by good sorption properties, accumulating compounds such as water or salts (acting as protection against moisture or saltiness), and can act as a carrier of active substances such as dyes or antibacterial and antifungal compounds. Inter alia antimicrobial characteristics can be obtained by an ion-exchange process. Silver is the most common ion used in the exchange process due to its stability and broad spectrum of antibacterial effect against *Escherichia coli*, *Bacillus subtilis*, *Staphylococcus aureus*, and *Pseudomonas aeruginosa* [6,7]. However, other metal ions can be used in the ion exchange procedure for the modification of zeolites to give them antibacterial properties [8,9]. Among others, both natural [10] and synthetic zeolites [5] exchanged with zinc and copper have shown an inhibitory effect against a wide range of bacterial strains. Silver-ion-exchanged zeolites were also found to be more effective against bacterial and candida species, while zinc zeolites exhibited noticeable antifungal properties [11].

The utilization of zeolite aggregates in the gypsum matrix is interesting and worth investigating because zeolites are not widely used in the production of gypsum binders and the available research shows that their use can bring immeasurable benefits. The literature shows that natural zeolite can act as an additive to plasters; however, there is no information on the use of synthetic ones. The tests showed very good physical and mechanical parameters of plasters with clinoptilolite (the most abundant natural zeolite), especially bonding and mechanical strength [12,13]. The addition of zeolite increases plasticity, stability after applying the plaster, vapor permeability, and resistance to fungi and algae [14,15].

The objective of the work was to determine the effect of zeolites (modified with transition metal ions) on the structure and properties of gypsum composites especially designed for use as biocidal plasters and mortars. Synthetic zeolite in the form of granules was selected for the tests. The zeolite granulate was obtained as a result of a hydrothermal reaction of expanded glass with an alkaline solution. The zeolite was activated by the ion exchange method—as a result, the Ni-, Cu-, and Zn-forms of the zeolites were obtained. The influence of synthetic zeolite on the properties of plaster was checked, and the antibacterial activity of Ni-, Cu-, and Zn-doped composites was investigated. Antibacterial activity tests were carried out against two reference strains of reference bacteria: Gram-negative *Escherichia coli* (ATCC 8739) and Gram-positive *Staphylococcus aureus* (ATCC 6538).

## 2. Materials and Methods

### 2.1. Preparation of Aggregate

**Figure 1:**
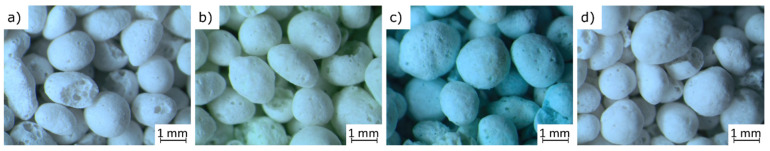
Zeolite lightweight aggregate as the reference sample analyzed (**a**) and impregnated with Ni^2+^ (**b**), Cu^2+^ (**c**), and Zn^2+^ ions (**d**).

The zeolite aggregate (Figure 1) was prepared according to the procedure previously described [16,17]. Expanded glass aggregates and sodium aluminate in a weight ratio of 9:1 was mixed with a 3 M NaOH solution and treated at 90 °C for 24 h. After this, the granules were washed with distilled water at a pH < 8 and dried.

In the impregnation procedure, the aggregate containing zeolite was mixed rotationally with an aqueous solution of appropriate metal salt in water (1 g of aggregate per 10 mL) for 24 h at 60 °C. Ni^2+^, Cu^2+^, and Zn^2+^ ions were introduced into the zeolite structure from aqueous solutions of nitrates. The concentration of all solutions was 0.1 mol/dm^3^. Twenty-four hours are a sufficient period of time to reach the equilibrium state between the metal ions in the solution and in the zeolite structure [18]. After this time, the aggregate was repeatedly rinsed with distilled water and dried.

### 2.2. Preparation of Gypsum Composites

The lightweight materials tested were prepared from gypsum (commercial mixture of synthetic and natural gypsum calcined to gypsum hemi-hydrate; 70% by mass), sand (0.25–0.5 mm fraction; 23%), lightweight aggregate (4%), lime (2.5%), and liquid agents. For the preparation of the reference sample, 4% expanded perlite with a fraction of 1–2 mm was used as a lightweight aggregate. Our previous research shows great similarity in the properties of plasters obtained with perlite and our proprietary zeolite aggregate.

For the preparation of the gypsum composite samples, the dry ingredients (gypsum, lime, and sand) were thoroughly mixed by hand. Then, water was added to obtain the water/binder ratio equal to 0.5 [12] and mixed in a standard mixer. Light aggregates were dosed as the last component of the mixture in line with industrial practice. Three types of samples were molded:

1. 25 × 25 × 100 mm bars for strength tests according to EN 13279-2:2014 standard;
2. A 75 × 75 × 15 mm plate for testing the thermal conductivity according to the EN 12667 standard;
3. 10 × 40 × 40 mm samples for antibacterial tests according to the ISO 22196:2011 standard.

After seven days of hardening in air, the samples were dried to a constant weight at 45 °C. Immediately before the tests, the samples were stabilized to air-drying conditions. Each type of sample was measured a minimum of six times.

### 2.3. Instrumentation

Solid state characterization techniques such as X-ray Fluorescence (XRF), X-ray diffraction (XRD), infrared spectroscopy (FT-IR), and scanning electron microscopy (SEM) were carried out both on raw materials and on the as-synthesized materials.

The chemical compositions of the starting materials were determined by X-ray fluorescence. The spectrum was detected using a wavelength dispersive X-ray fluorescence spectrometer (WD-XRF, PANalytical, Malvern, UK) Axios mAX 4 kW equipped with an Rh source. The Omnian standardless analysis package (PANalytical, Malvern, UK) was used for quantitative analysis of the spectra.

The resulting aggregates were analyzed in terms of the phase composition using the X-ray powder diffraction X’Pert system (PANalytical, Malvern, UK) and CuKα radiation. Measurements were carried out in the 2θ angle range of 5–90° for 2 h, with a step of 0.007. The phases were identified using an X’Pert HighScore Plus application (PANalytical, Malvern, UK) and the International Centre for Diffraction Data (Newtown Square, PA, USA).

The IR spectra shown in this work were obtained in diffuse reflectance on a Bruker Vertex 70 v spectrometer (Bruker, Billerica, MA, United States) using the standard transmission mode, and 128 scans were recorded over the range 4000–400 cm^–1^ at a nominal resolution of 4 cm^–1^. The background spectrum was collected using a pure KBr pellet (Merck, Darmstadt, Germany), and the spectra of samples were corrected with a linear baseline.

The scanning electron microscopy (SEM) observations were carried out on a Phenom XL system (Thermo Fisher Scientific, Waltham, MA, USA). The surface of the composite was sputter coated with gold, and an accelerating voltage of 10 kV was used during imaging. The microanalysis on the selected sections was performed on the same microscope equipped with EDX detectors (Thermo Fisher Scientific, Waltham, MA, USA). The acceleration voltage of the primary electron beam was set to 15 kV to ensure X-ray excitation for all relevant elements.

The pore structure of all specimens was characterized by a mercury intrusion porosimetry (MIP) method in the Poremaster 33 Quantachrome Instruments apparatus (Anton Paar GmbH, Graz, Austria), within a pressure range from 0.1 to 200 MPa.

The colony of bacteria grown on the media was counted using an automatic colony counter with Easy Count 2 model 7510/AES software (AES Chemunex, Marcy-l’Etoile, France) with statistical correction.

## 3. Results and Discussion

### 3.1. Physicochemical Properties of Zeolite Aggregate

The analysis of the aggregates obtained began with their visual observation (Figure 1). The color of the microspheres in visual assessment is favorable for the aggregate with sodium and zinc because it resembles the lighter types of perlites. Samples with nickel and copper deviate from market standards and may therefore be hardly accepted—although it should be noted that no discoloration related to the introduced colored aggregate was observed on the dried samples of gypsum composites.

The shape of the grains (oval or spherical) should be considered favorable from the point of view of applying and compacting the gypsum-based material.

**Figure 2:**
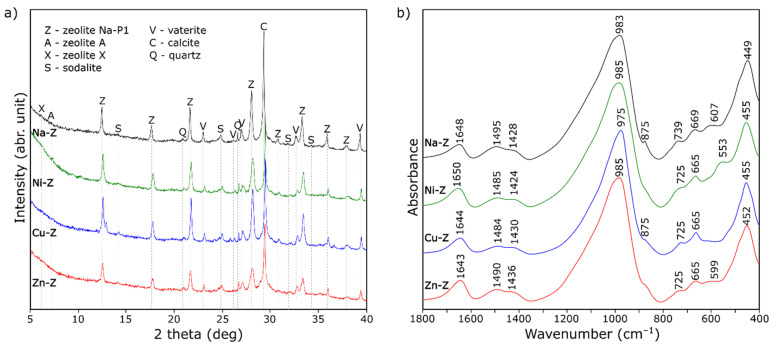
X-ray diffraction patterns (**a**) and FT-IR spectra (**b**) of lightweight aggregate containing zeolite impregnated with various ions.

The results of the mineralogical analysis of the analyzed aggregates are given in Figure 2a. The main component of the zeolite aggregate is the aluminosilicate amorphous phase (halo in the range of 20–40°) and the zeolite Na-P1. Small amounts of another zeolite phase, such as zeolite A and zeolite X, can also be detected. Hydroxy-sodalite and carbonates (calcite and vaterite) can be observed as a byproduct of synthesis. After the impregnation process, the zeolite phase loses its crystallinity (decrease in intensity and widening of the peaks), but the phase composition does not change. Slight shifts in the peak may be due to ion exchange-induced changes in unit cell parameters.

The FT-IR spectra of the aggregates doped with heavy metal ions are shown in Figure 2b. All of the spectra are quite similar. Visible differences can be observed when more detailed studies are carried out. In the case of Cu^2+^ ions, the position of the band with the highest integral intensity at about 985 cm^–1^ changes. This band can be assigned to asymmetric Si-O (Si, Al) vibrations and is a superposition of several component bands—it can come from both the zeolite phase and the amorphous phase. The shifting of this band toward lower wavenumbers may indicate both the modification of the zeolite structure and the depolymerization of the amorphous phase. In the case of the spectrum of the sample with nickel, the 553 cm^–1^ band has the highest intensity, which shows that this ion has the least influence on the structure of the discussed materials. Additionally, the IR results fully confirm the conclusions drawn from the X-ray diffraction pattern analysis.

Subsequently, to evaluate the elemental composition of the aggregate, XRF spectrophotometry was used—the results are collected in Table 1. As expected, the main elements of the zeolite aggregate were silica and alumina; alkali and alkaline earth metals were present in smaller amounts. Nickel, copper, and zinc, however, were almost not present in the initial material but were present only the after sorption processes. This confirmed the metal sorption into the zeolite structure.

**Figure 3:**
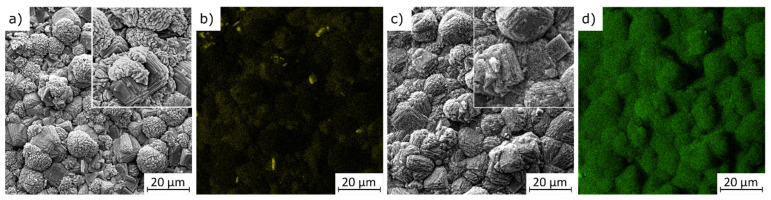
SEM images of zeolite aggregate doped with Cu^2+^ (**a**) and Zn^2+^ (**c**) ions and elemental distribution of copper (**b**) and zinc (**d**).

The morphology of zeolite Na-P1 resulting from partial zeolitization of the expanded glass aggregate by SEM and typical images is shown in Figure 3. Typical zeolite Na-P1 shapes, widely reported in other studies [19,20], were observed in all tests performed. The morphology of this aluminosilicate consists of secondary cauliflower-like aggregates, which are composed of smaller fragments formed by tetragonal crystals with well-defined edges and anisotropic crystal growth.

Both nickel and copper do not significantly influence the microstructure of the analyzed materials. These elements are evenly distributed in the zeolite structure. In Figure 3a, the SEM image shows the shape with a clear surface. The distribution of elements on the surface of the granules is uniform, with single precipitates visible in Figure 3b. On the other hand, Figure 3c shows the modification of the zeolite surface by zinc. The chemical composition of zinc-loaded zeolite is shown in Figure 3d.

### 3.2. Physical and Mechanical Properties of Gypsum Composites

**Figure 4:**
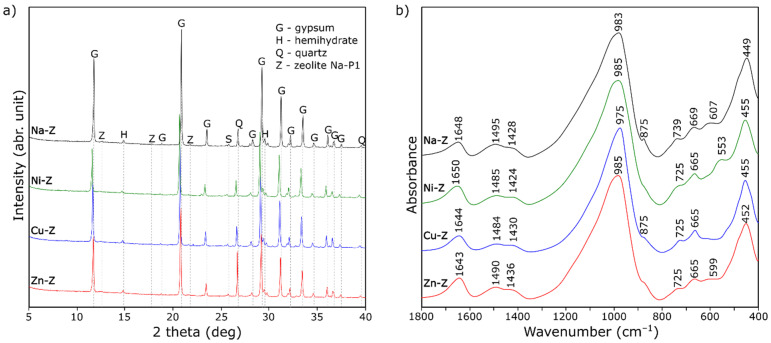
X-ray diffraction patterns (**a**) and FT-IR spectra (**b**) of gypsum composites with lightweight aggregates containing zeolite impregnated with various ions.

XRD patterns and FT-IR spectra of gypsum composites with lightweight aggregates containing zeolite impregnated with various ions are presented in Figure 4. No notable changes in the phase composition were identified for gypsum composites with aggregate doped with Ni^2+^, Cu^2+^, and Zn^2+^ ions compared with the Na-Z sample. The almost complete hydration of hemihydrates into dihydrates is evident from the XRD and FT-IR results obtained (Figure 4) for all composites. The peaks from the zeolite phase are of low intensity (due to the small amount of zeolite in relation to gypsum) but are noticeable. This is important information in the context of literature indications about the gradual disappearance of zeolite in the presence of lime [21,22]. Importantly, the tests repeated 6 months after forming the samples still show the presence of zeolite in the analyzed samples.

Table 2 presents the selected parameters describing the physical properties of the analyzed gypsum composites. The parameters for a typical lightweight material based on gypsum and expanded perlite are presented for comparative purposes. At first, gypsum composites with the addition of a zeolite aggregate are characterized by an increased density compared with the material with perlite, probably due to the increased absorbability of the filler. This observation is in line with our previous conclusions [23]. The resulting value for bulk density is around 1.05 g/cm^3^ and is typical for gypsum-based materials (usually greater than 0.8 g/cm^3^ [24]).

All of the composites analyzed exhibited good mechanical performance compared with other porous gypsum-based materials [25,26], including compared with those with perlite. Although flexural strength and hardness are at the same level, the addition of a zeolite aggregate doubles the compressive strength value. Because the flexural strength should be the result of the strength of the aggregate grains and their anchoring in the gypsum matrix, it can be assumed that the material analyzed is characterized by a good bond with the gypsum binder. The reason for this may be the already mentioned higher absorbability of the analyzed aggregate. The grains of this filler have a porous surface that induces the reinforcing effect of its connection with the matrix. Furthermore, the high pozzolanic activity of zeolites is known [27,28]; therefore, this phase could react with lime, enhancing this effect. It can be deduced that the use of a zeolite aggregate produces a reinforcing effect of gypsum composites compared with the commonly used expanded perlite.

**Figure 5:**
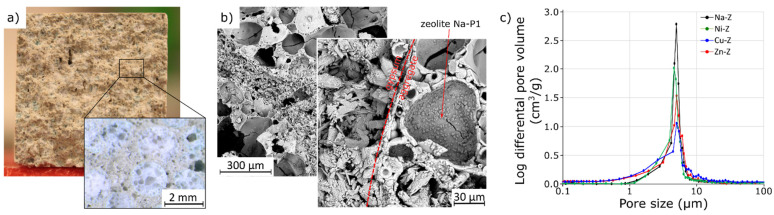
The analyzed fresh split surface of gypsum paste: photographs of plaster bar fractures with zeolite granulate (**a**), SEM microphotographs of relevant samples (**b**), and differential distribution curve of gypsum plasters measured by MIP (**c**).

The macro- and microscopic observations of the fractures of the samples are shown in Figure 5. The grains of the zeolite-doped filler behave similarly to perlite during the strength test; typically, the crack runs through these grains (Figure 5a). The morphological characteristics of the pastes were investigated on a fresh split surface using SEM (Figure 5b). A dense microstructure is observed in the volume of the gypsum paste. The dihydrate crystals form a compact matrix without pores or cracks. Particular grains of analyzed mineral aggregates are well bound with the surrounding calcium sulphate crystals—due to the high open porosity of the zeolite aggregate, the binder partially penetrates the pores.

The addition of ions with a potential antibacterial effect did not significantly affect the strength parameters of the samples (Table 2). Only the addition of copper appears to have a beneficial effect. The likely reason for this is the sealing of the aggregate-binder contact zone (also manifested in the increased density of this composite). It is also visible in the mercury intrusion porosimetry (MIP) results presented in Figure 5c. Small capillary pores (<1 μm) resulted in the evaporation of water from the binder fraction. Wider capillary pores (between 1 and 100 μm) were formed by the inter-granular space that is not completely filled by the binder. In Figure 5c, it is clearly seen that the composites analyzed contained only capillary pores [29], and the smallest volume was obtained as a result of the analysis of the composite with copper.

Gypsum binders are characterized by a high sensitivity to water, and therefore, the influence of the aggregates used on this factor was checked. The results—water absorption (after 24 h of immersion in water) and softening factor (in the compressive strength test)—are presented in Table 2. The aggregate analyzed is undoubtedly an absorbable aggregate, but compared with perlite, this value is much lower, especially for the sample with an admixture of copper. Water soaking is a process determined by diffusion and capillary rise, in which both gypsum and aggregate are involved. The zeolite aggregate, despite absorbing water, does not participate in its transport to the same extent as perlite. There are three possible reasons for this. First, in the structure of the microstructure of zeolite granules, they dominate spherical pores with dimensions exceeding the optimum for rising capillary action. Second, effectively bonding the aggregate with the gypsum binder (see Figure 5) contributed to the reduction in porosity in the aggregate–gypsum contact zone and therefore affected water transport. Finally, the effect of zeolite addition on water migration is also known [15].

One of the advantages of lightweight gypsum products is their low thermal conductivity. Aggregates with the addition of zeolite, which in some of the presented studies showed similarity to perlite, have a density that is several times higher, which is reflected in thermal conductivity. The open porosity of the granules can also be a problem, favoring the absorption of the binder by the granules. These values, however, are still significantly higher than those for typical gypsum plasters, for which this value is approximately 0.35 W/(m·K) [30].

### 3.3. Antimicrobial Properties of Gypsum Composites

**Figure 6:**
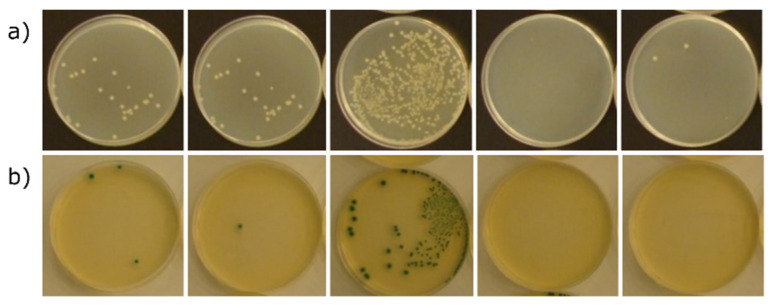
Growth of *Escherichia coli* on PCA (**a**) and TBX (**b**) media after culturing the suspension samples recovered from the test materials after 24 h of incubation under optimal growth conditions. From left to right: perlite, Na-Z, Ni-Z, Cu-Z, and Zn-Z.

**Figure 7:**
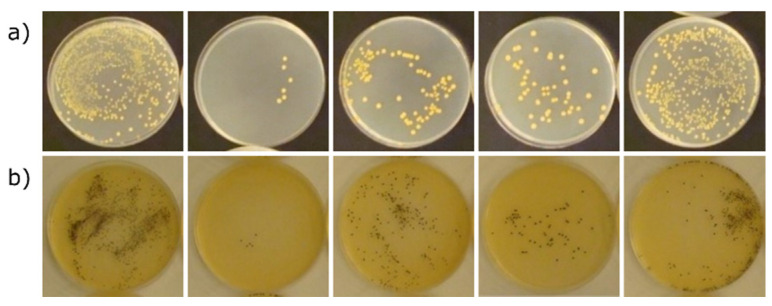
Growth of *Staphylococcus aureus* on PCA (**a**) and Baird–Parker (**b**) media after culturing the suspension samples recovered from the test materials after 24 h of incubation under optimal growth conditions. From left to right: perlite, Na-Z, Ni-Z, Cu-Z, and Zn-Z.

The evaluation of antibacterial activity according to the methodology used in this study is based on the determination of the degree of reduction in the number of two reference bacteria strains (*Escherichia coli* and *Staphylococcus aureus*) during 24 h contact of the tested material with the antibacterial component. For this purpose, the suspension of bacteria in the nutrient medium is introduced to the surface of the determined sample and incubated under optimal conditions. It should be noted that this method is not dedicated to porous materials—soaking the inoculum of microorganisms into the tested material may prevent full recovery of the suspension and, thus, cause an error in the interpretation of the antibacterial effect. The results of determining the reduction in the number of bacteria as a result of 24 h contact with the tested materials are presented in Table 3. Documentation of the growth of bacterial colonies on the culture medium on the basis of which the calculations were made is presented in Figure 6 and Figure 7.

The values of the degree of reduction in the number of *Escherichia coli* bacteria as a result of 24 h contact with the tested materials (Table 3) are insufficient to fully confirm their antibacterial activity against this bacteria, although in the case of material with zinc and copper, the degree of reduction was close to the ISO 22196:2011 standard value adopted for the recognition of antimicrobial activity, equal to 2, and amounted to 1.23 for Zn-Z and 1.68 for Cu-Z series. The values of the degree of reduction in the number of *Staphylococcus aureus* bacteria as a result of 24 h contact with the tested materials, as in the case of *Escherichia coli*, are not sufficient to fully confirm their antibacterial activity. The highest degree of reduction in the number of *Staphylococcus aureus* bacteria was recorded for materials with sodium and copper, where it was 1.98 and 1.42, respectively. None of the tested materials resulted in a reduction in the number of bacteria exceeding 2 on a logarithmic scale, which was therefore sufficient to recognize their antibacterial properties in accordance with the recommendations of the methodology used. It should be noted that incomplete confirmation of the antibacterial effect of the tested materials could have been influenced by the porosity of the surface. Strong absorption of the bacterial suspension after inoculation of the samples made it difficult to rinse the bacteria from the blocks and to correctly determine their number, which is confirmed by the failure to meet the validation conditions of the method. On the other hand, microscopic examinations made it possible to find the absence of bacteria inside and even on the surface of the granules in the case of copper and zinc ions, which makes us believe that both materials are at least bacteriostatic.

It is clear from the results presented in Figure 6 and Figure 7 that the antibacterial properties vary directly with the type of ion. All of these data confirmed that Cu–Z had an obvious advantage in antibacterial properties compared with other as-prepared materials. The beneficial effect of zinc ions against *Escherichia coli* can also be observed. The high antibacterial effect of Zn-zeolite and Cu-zeolite is in agreement with the study previously reported [31,32]. Other authors also ruled out the antimicrobial effect of nickel-doped zeolite [9]. The effect of the sodium form of zeolite on the growth of *Staphylococcus aureus* is somewhat surprising; however, it is known that the bactericidal activity of zeolites containing bioactive metals is due not only to the release of ions of these metals into the surrounding liquid medium but also to the zeolite matrix itself.

For both *Escherichia coli* and *Staphylococcus aureus*, the degree of reduction was greater for compounds with Cu^2+^ ions than Zn^2+^. This is in line with the data from the literature [31,32,33]. The mechanisms of action of the Cu^2+^ and Zn^2+^ ions differ. It is assumed that Zn^2+^ mainly restricts the essential Mn^2+^ uptake by bacterial cells and causes their death [32,34] while Cu^2+^ damages microbial DNA [35]. This is probably because the active metal zinc is more stable in the state of Zn^2+^, while Cu^2+^ is more easily reduced to the low-valent state.

The results of the tests also showed that *Staphylococcus aureus* is more resistant to the composites doped with Zn^2+^. This is also consistent with the results of a previous study [32,36]. Gram-positive bacteria have a thicker peptidoglycan layer and the predominant mechanism of inhibiting *Staphylococcus aureus* bacteria can be attributed to the induction of the downregulation of amino acid synthesis and the dysfunction of vital bacterial enzymes [37].

## 4. Conclusions

On the basis of the results obtained, the following conclusions can be drawn:

1. The color of the aggregate is lighter than that of the typical gypsum binder; use may be limited by the color variants of the aggregate.
2. The aggregates assessed showed good embedding of the grains in the gypsum binder matrix.
3. Based on the method of breaking, the zeolite aggregate showed a lower strength than that of the gypsum binder. The effect of introducing active ions into the zeolite structure is negligible.
4. Gypsum composites based on the zeolite aggregate are characterized by an appropriate strength, reduced density, and a thermal conductivity that is appropriate for gypsum plasters. Its properties resemble those of perlite. The proposed aggregate was evaluated as being useful in building materials technologies, both for small-size cast elements and for plaster.
5. The antibacterial activity tests conducted against two reference strains of *Staphylococcus aureus* and *Escherichia coli* bacteria did not fully confirm the antibacterial effectiveness of the tested formulations after their incorporation into the plaster layer. On the other hand, a noticeable bacteriostatic effect was found for the formulations with copper and zinc against *Escherichia coli* and with copper against *Staphylococcus aureus.*
6. The research carried out also revealed a certain gap in the methodology of antibacterial determinations related to the analyses carried out on porous materials.

## Figures and Tables

**Table 1:** Chemical compositions (wt. %) of the aggregates.

Sample	SiO_2_	TiO_2_	Al_2_O_3_	Fe_2_O_3_	MgO	CaO	Na_2_O	K_2_O	NiO	CuO	ZnO	Res.
Na-Z	47.80	0.32	12.59	1.56	2.90	25.51	7.88	0.13	0.07	0.06	0.16	1.02
Ni-Z	45.35	0.24	10.10	1.28	3.12	21.16	3.19	0.15	**14.22**	0.06	0.15	0.98
Cu-Z	41.92	0.24	9.31	1.33	3.04	21.04	2.13	0.13	0.00	**19.15**	0.12	1.59
Zn-Z	45.18	0.28	9.63	1.26	3.18	20.91	3.99	0.15	0.30	0.30	**14.25**	0.57

**Table 2:** Physical properties of gypsum composites.

Sample	Bulk Density, (g/cm^3^)	Flexural Strength, (MPa)	Compressive Strength, (MPa)	Hardness, (MPa)	Water Absorption, (%)	Softening Factor	Thermal Conductivity, (W/(m·K))
Na-Z	1.05 ± 0.01	1.07 ± 0.06	4.04 ± 0.72	8.1 ± 1.0	27.32	0.48 ± 0.02	0.313
Ni-Z	1.05 ± 0.01	1.24 ± 0.06	4.83 ± 0.26	7.0 ± 0.9	27.20	0.49 ± 0.02	0.293
Cu-Z	1.10 ± 0.02	1.86 ± 0.09	5.54 ± 0.41	8.3 ± 0.7	25.97	0.44 ± 0.02	0.299
Zn-Z	1.06 ± 0.01	0.98 ± 0.05	4.65 ± 0.80	8.6 ± 0.6	27.15	0.50 ± 0.02	0.305
perlite	0.91 ± 0.01	1.08 ± 0.06	2.93 ± 0.18	6.8 ± 1.2	31.38	0.46 ± 0.02	0.236

**Table 3:** Values of the reduction in the number of bacteria as a result of 24 h contact with the tested materials—antibacterial activity according to the ISO 22196:2011 standard.

Sample	*Escherichia coli*	*Staphylococcus aureus*
Na-Z	0.22	1.98
Ni-Z	0.42	0.98
Cu-Z	1.68	1.42
Zn-Z	1.23	0.72

## Data Availability

The data presented in this study are available from the corresponding author upon request.

## Sample Availability

Samples of the zeolite-supported aggregate are available from the authors.
